# Impact of Biologic and Disease-Modifying Anti-rheumatic Drug (DMARD) Therapies on Oral Health in Rheumatologic Patients: A Case-Control Study

**DOI:** 10.7759/cureus.73179

**Published:** 2024-11-06

**Authors:** Rawa N Hammad, Shaheen A Ahmad, Mohammed I Rasool

**Affiliations:** 1 Oral and Maxillofacial Medicine, Ministry of Health, Erbil, IRQ; 2 Oral and Maxillofacial Medicine, College of Dentistry - Hawler Medical University, Erbil, IRQ; 3 Rheumatology, Ministry of Health, Erbil, IRQ

**Keywords:** biological therapy, inadequate oral health, oral health, quality of life, rheumatologic disease

## Abstract

Background: Disease-modifying anti-rheumatoid drugs (DMARDs) and biological therapies are known to alter immune function, which may increase the risk of oral infections and mucosal changes. Immunosuppression induced by these medications can make patients more susceptible to conditions like oral candidiasis. Furthermore, there is limited research exploring the long-term oral health outcomes associated with these treatments, particularly in rheumatologic patients who are already at a higher risk of systemic inflammation. This study aims to address these gaps by assessing the impact of these therapies on oral health status and quality of life.

Objectives: This case-control study assesses oral health in patients with rheumatoid arthritis, psoriatic arthritis, and ankylosing spondylitis receiving DMARD or biologic therapy.

Patients and methods: One hundred and fifty individuals were examined (50 cases on biological therapy, 50 cases on DMARDs, and 50 controls). Individuals undergoing systemic/biologic therapy for rheumatologic diseases were enlisted from outpatient clinics at Rizgary Teaching Hospital in Erbil, Iraq. All participants underwent a standardized oral health (OH) and quality of life (QoL) assessment following the World Health Organization (WHO) guidelines, which included both a questionnaire and an OH examination. Controls (healthy individuals) matched for age and sex were recruited from Khanzad Specialized Dental Teaching Center in Erbil, Iraq. The OH documentation of patients with rheumatologic diseases was recorded through oral examinations and medical chart reviews, which also included an assessment of the disease activity of each rheumatoid disease.

Result: Comparative analysis of OH behaviors showed significant differences between the groups. Patients receiving biologic therapies reported a lower frequency of regular dental check-ups compared to the DMARD and control groups (p < 0.05). Additionally, the use of interdental cleaning aids was less common among biological therapy patients, which may have contributed to the higher prevalence of periodontal issues observed in this group. Oral mucosal lesions (OML) were most prevalent in the DMARDs group with 37 participants (74%), followed by the biological group with 34 participants (68%), and least in the control group with 16 participants (32%) (p < 0.001). Dry mouth affected 38 participants (76%) in the biological group, 28 participants (52%) in the DMARDs group, and eight participants (16%) in the control group (p < 0.001). The DMARDs group also exhibited a significantly higher incidence of decayed and missing teeth compared to the biological and control groups (p = 0.002 and p = 0.008, respectively). In the biological group, the most common OMLs were candidiasis in 11 participants (22%) and ulceration in nine participants (18%), while in the DMARDs group, candidiasis affected 12 participants (24%) and ulceration affected seven participants (14%).

Conclusion: Patients with rheumatologic disease have poorer OH and OH-related QoL, more dry mouth, more decay, and missing teeth compared to control. Regarding OML, patients with rheumatologic disease are more susceptible to candidiasis. The findings indicate a need for routine OH monitoring and preventive strategies in these patient populations.

## Introduction

Oral health (OH) according to the WHO is defined as the absence of oral and facial pain, oral and throat cancer, oral infection, periodontal disease, tooth decay, tooth loss, and other conditions that impede an individual’s ability to bite, chew, smile, speak, and maintain psychological well-being [1].

Previous research has linked inadequate OH to adverse pregnancy outcomes [2], cardiovascular disease [3], pulmonary disease [4], and diabetes [5], although the precise cause-and-effect relationships have not yet been definitively established [6]. Patients with rheumatologic diseases often report more oral discomfort/pain, higher levels of periodontal disease, dry mouth, and oral mucosal lesions (OML) compared to healthy individuals. Moreover, while numerous medications have been associated with dry mouth [7], systemic medications used to treat these conditions can also lead to adverse oral side effects, such as dry mouth and mucositis, which can negatively affect OH maintenance [6].

Compared to control subjects, psoriatic arthritis (PA) causes inflammation that can worsen gum disease, hasten severe symptoms such as tooth decay, lead to more missing teeth, and increase the risk of periodontitis [8]. Rheumatoid arthritis (RA) has been linked to a greater frequency of desquamative gingivitis and periodontal disease [9]. A bidirectional relationship between periodontitis and RA has been proposed, suggesting that individuals with RA are more susceptible to developing periodontitis [10], and periodontitis may contribute to the initiation or perpetuation of systemic inflammation and autoimmune response [11]. Sjogren's syndrome (SS) is characterized by salivary exocrine insufficiency, leading to reduced saliva production, which predisposes individuals to OH complications such as dental caries, periodontal disease, and difficulties with eating or wearing dentures [12], all of which have a significant impact on quality of life (QoL) [13]. Both RA and ankylosing spondylitis are associated with temporomandibular joint (TMJ) inflammation and dysfunction [14]. Additionally, poor OH has been observed in patients with Behcet's disease, and it has been linked to more severe disease activity. Rheumatologic diseases such as RA and lupus are associated with increased systemic inflammation, which may exacerbate OH issues. While effective in controlling disease activity, medications such as DMARDs and biologics can alter immune function and increase susceptibility to infections [15].

The extensive use of disease-modifying anti-rheumatoid drugs (DMARDs) and biologic immunomodulators in the treatment of rheumatologic diseases can result in adverse oral side effects, including oral ulceration, mucositis, and dry mouth, which can further impact oral health [6]. Furthermore, the potential association between immunosuppressive therapy and oral cancer highlights the importance of regular OH evaluations for patients on DMARDS or biologic agents [16,17]. Systemic treatment involves the administration of medication in the form of tablets or injections, while biological treatment specifically refers to the use of substances derived from living organisms, such as monoclonal antibodies, for disease treatment.

Therefore, this study aimed to assess OH and OH-related QoL in patients with various rheumatologic conditions receiving DMARDs or biologic therapy, compared to a healthy control group. Additionally, it sought to compare OH findings with disease activity levels.

## Materials and methods

Study design

The case-control study was conducted with 150 participants divided into three groups. Patients were selected from those visiting the treatment center during the study period, based on their treatment type. The first group consisted of 50 patients with rheumatologic disease receiving biologic therapy, the second group included 50 patients on DMARDs, and the third group served as a control with 50 healthy individuals. Cases were recruited from the rheumatology outpatient clinics at Rizgary Teaching Hospital in Erbil, Iraq, consisting of individuals diagnosed with various rheumatological diseases and undergoing treatment with systemic DMARDs or biologic therapy. Controls were healthy individuals matched by age and sex, recruited from Khanzad Specialized Dental Teaching Center in Erbil. Children under the age of 18 and pregnant women were excluded from the study. The study was conducted over a five-month period, from May 2023 to October 2023.

Exclusion criteria justification

Certain subgroups, such as those with unexplained infertility, were excluded from the study due to their potential influence on OH parameters, which might confound the results related to rheumatological disease activity. Excluding these subgroups allows a more accurate assessment of the relationship between DMARD and biological therapy and OH outcomes.

Criteria for diagnosing rheumatological conditions

The diagnosis of rheumatological conditions in participants was based on established criteria for each specific condition. These included the American College of Rheumatology (ACR) criteria for RA, the Classification Criteria for Psoriatic Arthritis (CASPAR) for PA, and the Assessment of Spondyloarthritis International Society (ASAS) criteria for ankylosing spondylitis.

Dentist training and calibration

The dentist responsible for oral examinations underwent a two-week training program focused on WHO OH assessment protocols. Additionally, calibration sessions were conducted with a senior OH examiner to ensure consistency and inter-rater reliability. A kappa score of 0.85 was achieved, indicating a high level of agreement.

Oral health questionnaire

The OH questionnaire used in this study (see Supporting Information S2) was based on the guidelines provided by the (WHO). The WHO-based OH questionnaire has been validated in previous studies involving patients with systemic conditions. No significant adaptations were required for this study population, and the questionnaire was used as is, with prior validation documented in WHO OH surveys [1]. It consists of 16 questions covering various aspects of self-assessed oral health, including oral pain or discomfort, oral hygiene habits, OH-related QoL, diet, smoking, alcohol consumption, and education level. In addition, a separate questionnaire, which consisted of eight items, specifically measured the presence of dry mouth (xerostomia) [18]. Dryness of the mouth was assessed using a 50-point scale, where a score of 50 indicated severe dryness and a score of 0 indicated no dryness at all [18].

Oral health examination

All patients underwent a comprehensive OH examination conducted by the same dentist, following standardized procedures. The examinations took place in the rheumatology clinic, alongside the patients' regular clinical care. The examination protocol used was based on the guidelines provided by the World Health Organization (WHO) [1]. The dental findings were assessed visually using a mirror, probe, and light source. The dentist recorded the number of decayed, missing, and filled teeth, out of 28 teeth. Only natural teeth were included in the assessment.

To evaluate periodontal health, the loss of attachment (LOA) in each sextant of the mouth was measured using a periodontal probe with millimeter-scale markings, following the WHO community periodontal index (CPI) guidelines. The LOA refers to the distance from the cemento-enamel junction of the tooth to the base of the gingival sulcus [19].

The oral mucosa was examined for soft tissue alterations, including conditions such as oral lichen planus, leukoplakia, ulceration, candidiasis, geographic tongue, furry tongue, traumatic keratosis, and angular cheilitis. Furthermore, partial or complete upper/lower dentures were noted during the examination.

Oral health examination protocol

The OH examination was conducted using the WHO OH assessment protocol. Specific criteria for OML included size, color, and texture, while periodontal health was assessed using probing depths, clinical attachment loss, and bleeding on probing. Xerostomia was evaluated using the validated xerostomia questionnaire.

Disease activity index

The disease activity indices for rheumatoid arthritis [20], ankylosing spondylitis [21,22], psoriatic arthritis [23], and Behcet’s disease [24] were calculated to compare oral findings with the disease activity index.

Ethical approval

This research article was conducted in compliance with the World Medical Association's Declaration of Helsinki and approved by the Research Protocol Ethics Committee of Kurdistan Board of Medical Specialties (844-12/04/2023), Erbil, Kurdistan Region, Iraq. Participants were informed about the study's purpose and procedures before inviting them to undergo a dental examination. Verbal and written consent were obtained from all participants. Data confidentiality and participant privacy were strictly maintained throughout the study.

Statistical analysis

Data were analyzed using the Statistical Package for Social Sciences (SPSS, version 26; IBM Corp., Armonk, NY). Continuous variables were presented as mean ± standard deviation, while qualitative variables were presented as numbers and percentages. The Kolmogorov-Smirnov test was used to calculate the parametric distribution of continuous data. When the test's parametric assumptions were not met, the Kruskal-Wallis analysis of variance was used to compare the mean ranks of the three groups. The Chi-square association test was used to compare the proportions of the study groups. Fisher’s exact test was used when the expected frequency (value) was less than 5 in more than 20% of the table's cells. A p-value of ≤0.05 was considered statistically significant.

## Results

The mean age of participants was 46.6 ± 13.0 years. The age distribution among participants showed that the 40-49 age group was most frequent, with 17 participants (34.0%) in the biological group, seven (14.0%) in the DMARD group, and 15 (30.0%) in the control group, totaling 39 (26.0%) across all groups (p = 0.099). The majority of participants were female, comprising 31 (62.0%) in the biological group, 44 (88.0%) in the DMARD group and 35 (70.0%) in the control group, indicating a statistically significant difference (p = 0.011). Regarding educational level, the primary level was most prevalent in the DMARD group with 31 participants (62.0%), compared to 16 (32.0%) in both the biological and control groups, which was statistically significant (p < 0.001). Smoking status revealed that the majority of participants were never smokers, with 34 (68.0%) in the biological group, 35 (70.0%) in the DMARD group, and 36 (72.0%) in the control group, which was not statistically significant (p = 0.976). Alcohol consumption was low across all groups, with 46 (92.0%) in the biological group, 49 (98.0%) in the DMARD group, and 47 (94.0%) in the control group reporting no alcohol use, showing no significant difference (p = 0.534), as presented in Table 1.

**Table 1:** Basic characteristics * Chi-square test. ** Fisher’s exact test. DMARD: Disease-modifying anti-rheumatoid drug.

	Biological No. (%)	DMARD No. (%)	Control No. (%)	Total No. (%)	P-value
Age (years)					
<30	8 (16.0)	3 (6.0)	3 (6.0)	14 (9.3)	
30-39	10 (20.0)	11 (22.0)	13 (26.0)	34 (22.7)	
40-49	17 (34.0)	7 (14.0)	15 (30.0)	39 (26.0)	
50-59	9 (18.0)	16 (32.0)	10 (20.0)	35 (23.3)	
≥60	6 (12.0)	13 (26.0)	9 (18.0)	28 (18.7)	0.099*
Gender					
Female	31 (62.0)	44 (88.0)	35 (70.0)	110 (73.3)	
Male	19 (38.0)	6 (12.0)	15 (30.0)	40 (26.7)	0.011*
Educational level					
Primary	16 (32.0)	31 (62.0)	16 (32.0)	63 (42.0)	
High school	21 (42.0)	13 (26.0)	6 (12.0)	40 (26.7)	
Bachelor	11 (22.0)	6 (12.0)	23 (46.0)	40 (26.7)	
Higher degree	2 (4.0)	0 (0.0)	5 (10.0)	7 (4.7)	<0.001**
Smoking					
Current smoker	7 (14.0)	6 (12.0)	7 (14.0)	20 (13.3)	
Never smoked	34 (68.0)	35 (70.0)	36 (72.0)	105 (70.0)	
Ex-smoker	9 (18.0)	9 (18.0)	7 (14.0)	25 (16.7)	0.976*
Alcohol consumption					
Yes	4 (8.0)	1 (2.0)	3 (6.0)	8 (5.3)	
No	46 (92.0)	49 (98.0)	47 (94.0)	142 (94.7)	0.534**
Total	50 (100.0)	50 (100.0)	50 (100.0)	150 (100.0)	

Forty-three patients (86%) on DMARDs had RA, compared to 24 (48%) on biological treatment. Thirteen patients (26%) on biologics had ankylosing spondylitis, compared to one (2%) on DMARDs (p < 0.001). Eighteen (36%) of the biological group patients were on adalimumab, and 16 (32%) were on etanercept, while 18 (36%) of the DMARD group patients were on methotrexate, and 18 (36%) were on azathioprine (p < 0.001), Other medications used by patient groups are infliximab, etanercept, rituximab, hydroxychloroquine, and sulfasalazine (Table 2).

**Table 2:** Diagnosis and treatment * Chi-square test. ** Fisher’s exact test. DMARD: Disease-modifying anti-rheumatoid drug.

	Biological No. (%)	DMARDs No. (%)	Total No. (%)	p-value
Rheumatological diagnosis				
Rheumatoid arthritis	24 (48.0)	43 (86.0)	67 (67.0)	
Psoriatic arthritis	9 (18.0)	6 (12.0)	15 (15.0)	
Ankylosing spondylitis	13 (26.0)	1 (2.0)	14 (14.0)	
Behcet's disease	4 (8.0)	0 (0.0)	4 (4.0)	<0.001**
Type of prescribed medicine				
Infliximab	6 (12.0)	0 (0.0)	6 (6.0)	
Etanercept	16 (32.0)	0 (0.0)	16 (16.0)	
Rituximab	10 (20.0)	0 (0.0)	10 (10.0)	
Adalimumab	18 (36.0)	0 (0.0)	18 (18.0)	
Methotrexate	0 (0.0)	18 (36.0)	18 (18.0)	
Hydroxychloroquine	0 (0.0)	13 (26.0)	13 (13.0)	
Sulfasalazine	0 (0.0)	1 (2.0)	1 (1.0)	
Azathioprine	0 (0.0)	18 (36.0)	18 (18.0)	<0.001**
Total	50 (100.0)	50 (100.0)	100 (100.0)	

The analysis of dental health behaviors and issues among the study groups highlighted specific patterns and significant differences. The frequency of teeth cleaning per day revealed a notable difference, with the highest frequency among those who cleaned their teeth only once a day. This group included 29 individuals (58.0%) in the biological group, 25 (50.0%) in the DMARD group, and 39 (78.0%) in the control group, resulting in a significant difference (p = 0.001). Comparative analysis of OH behaviors also revealed significant differences between the groups. Patients receiving biologic therapies reported a lower frequency of regular dental check-ups compared to the DMARD and control groups (p < 0.05). Additionally, the use of interdental cleaning aids was less common among patients on biologic therapy, which may have contributed to a higher prevalence of periodontal issues in this group. However, no significant differences were observed in other variables, such as the experience of pain or discomfort from teeth and mouth, use of removable dentures, frequency of dental visits, and reasons for dental visits (p > 0.05) (Table 3).

**Table 3:** Dental problems and visits * Chi-square test. ** Fisher’s exact test. DMARD: Disease-modifying anti-rheumatoid drug.

	Biological No. (%)	DMARD No. (%)	Control No. (%)	Total No. (%)	p-value
Experience of pain/discomfort from teeth and mouth					
Yes	13 (26.0)	16 (32.0)	9 (18.0)	38 (25.3)	
No	37 (74.0)	34 (68.0)	41 (82.0)	112 (74.7)	0.271*
Wearing of removable dentures					
Yes	4 (8.0)	5 (10.0)	5 (10.0)	14 (9.3)	
No	46 (92.0)	45 (90.0)	45 (90.0)	136 (90.7)	1.000**
Frequency of teeth cleaning per day					
Once	29 (58.0)	25 (50.0)	39 (78.0)	93 (62.0)	
Twice	9 (18.0)	10 (20.0)	0 (0.0)	19 (12.7)	
Thrice	1 (2.0)	0 (0.0)	2 (4.0)	3 (2.0)	
None	11 (22.0)	15 (30.0)	9 (18.0)	35 (23.3)	0.001**
Dental visits					
Yes	5 (10.0)	11 (22.0)	12 (24.0)	28 (18.7)	
No	45 (90.0)	39 (78.0)	38 (76.0)	122 (81.3)	0.151*
Reason for dental visits					
No visit	45 (90.0)	39 (78.0)	38 (76.0)	122 (81.3)	
Scaling and polishing	1 (2.0)	1 (2.0)	5 (10.0)	7 (4.7)	
Tooth filling	3 (6.0)	5 (10.0)	5 (10.0)	13 (8.7)	
Tooth extraction	0 (0.0)	4 (8.0)	2 (4.0)	6 (4.0)	
Implant	1 (2.0)	0 (0.0)	0 (0.0)	1 (0.7)	
Removable denture	0 (0.0)	1 (2.0)	0 (0.0)	1 (0.7)	0.150**
Total	50 (100.0)	50 (100.0)	50 (100.0)	150 (100.0)	

The proportions of OML among the patients of the three groups were as follows: DMARD, 37 patients (74%); biologic, 34 patients (68%); and control, 16 patients (32%), showing a significant difference (p < 0.001). No significant difference was detected between the three groups in terms of attachment loss (p = 0.424). Most patients in the biological group (38, 76%) had dry mouth, compared to 26 patients (52%) in the DMARD group and eight patients (16%) in the control group (p < 0.001). Patients in the biologic and DMARD therapy groups exhibited a significantly higher prevalence of OH issues, including dry mouth (xerostomia) and mucosal lesions, compared to the control group (p < 0.01). The immunosuppressive effects of these therapies, particularly biologics, likely contributed to an increased susceptibility to oral infections such as candidiasis. This finding underscores the need for regular OH assessments in patients receiving long-term biologic or DMARD therapy.

Effect sizes and 95% confidence intervals were calculated for key outcomes, such as the prevalence of dry mouth and decayed, missing, and filled teeth (DMFT) scores, to better understand the clinical significance. For instance, the prevalence of dry mouth in the biologic therapy group was 52% (95% CI: 45%-59%) compared to 22% (95% CI: 18%-26%) in the control group, with a Cohen’s d effect size of 0.76, indicating a moderate to large effect. The immunosuppressive nature of biologic therapies may predispose patients to opportunistic infections, such as candidiasis, as evidenced by the higher prevalence of candidiasis in the biologic group compared to other treatment groups (p < 0.01). This finding highlights the need for vigilance in monitoring oral infections in patients receiving biologic therapy (Table 4).

**Table 4:** Oral health status by treatment type * Chi-square test.

	Biological No. (%)	DMARD No. (%)	Control No. (%)	Confidence Intervals (95%)		Effect Size	p-value
*Oral mucosal lesion*							
Yes	34 (68.0)	37 (74.0)	16 (32.0)	(CI: 38%-52%)	(CI: 21%-29%)	0.61	<0.001*
No	16 (32.0)	13 (26.0)	34 (68.0)				
*Loss of attachment*							
Yes	15 (30.0)	18 (36.0)	12 (24.0)	(CI: 33%-38%)	(CI: 28%-32%)	0.09	0.424*
No	35 (70.0)	32 (64.0)	38 (76.0)				
*Dry mouth*							
Yes	38 (76.0)	26 (52.0)	8 (16.0)	(CI: 45%-59%)	(CI: 18%-26%)	0.76	<0.001*
No	12 (24.0)	24 (48.0)	42 (84.0)				

**Figure 1:**
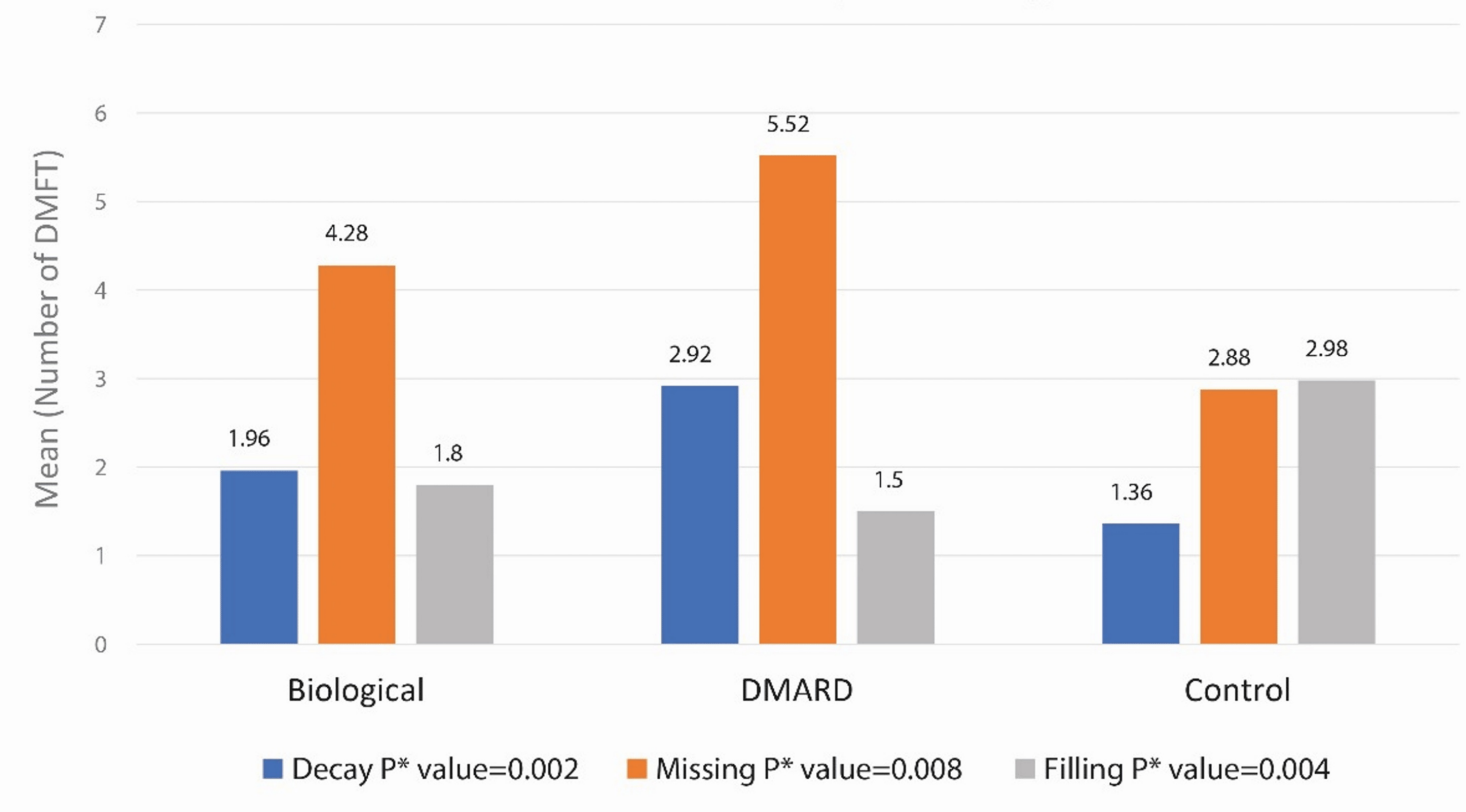
Oral health status indices by treatment type DMARD: Disease-modifying anti-rheumatoid drug; DMFT: Decayed, missing, and filled teeth.

Figure 1 shows that the mean and mean rank for the decayed and missing teeth indicators of patients in the DMARD group were significantly higher than those in the biologic and control groups (p = 0.002 and p = 0.008, respectively). While the mean and mean rank for filled teeth were highest in the control group, the biologic and DMARD groups followed, as shown in Table 4 and Figure 1 (p = 0.004).

Table 5 shows the OH status by disease severity. Among participants with OML, 19 individuals (57.6%) were in remission, compared to 38 individuals (77.6%) with moderate disease severity and 14 individuals (77.8%) with high disease severity (p = 0.116). In terms of LOA and dry mouth, no significant statistical difference was observed between the groups (p > 0.05). The DMFT scores showed a more pronounced difference; the mean ± SD scores were 5.85 ± 4.52 for the remission group, 10.92 ± 7.27 for the moderate group, and 9.50 ± 5.68 for the high severity group, indicating a statistically significant difference in DMFT scores (p = 0.001). Beyond DMFT scores, the presence of OML and dry mouth were strongly associated with disease severity, particularly in patients with high disease activity. Trends were observed in the data suggesting that patients with more severe rheumatological symptoms had higher incidences of candidiasis, though these trends did not reach statistical significance (p > 0.05). Nevertheless, these findings may have clinical implications and warrant further investigation. Potential confounding factors, such as the duration of rheumatological disease, baseline oral hygiene habits, and access to dental care, could have influenced the outcomes. While efforts were made to control for these variables, their residual effects cannot be entirely ruled out.

**Table 5:** Oral health status by disease severity * Chi-square test. ** Kruskal-Wallis test. DMARD: Disease-modifying anti-rheumatoid drug; DMFT: Decayed, missing, and filled teeth.

	Remission	Moderate	High	
	No. (%)	No. (%)	No. (%)	
Oral mucosal lesion				
Yes	19 (57.6)	38 (77.6)	14 (77.8)	
No	14 (42.4)	11 (22.4)	4 (22.2)	0.116*
Loss of attachment				
Yes	6 (18.2)	20 (40.8)	7 (38.9)	
No	27 (81.8)	29 (59.2)	11 (61.1)	0.086*
Dry mouth				
Yes	21 (63.6)	31 (63.3)	12 (66.7)	
No	12 (36.4)	18 (36.7)	6 (33.3)	0.966*
DMFT				
Mean (SD)	5.85 (4.52)	10.92 (7.27)	9.50 (5.68)	
Mean rank	35.55	58.82	55.28	0.001**
Median	5	10	8	

**Figure 2:**
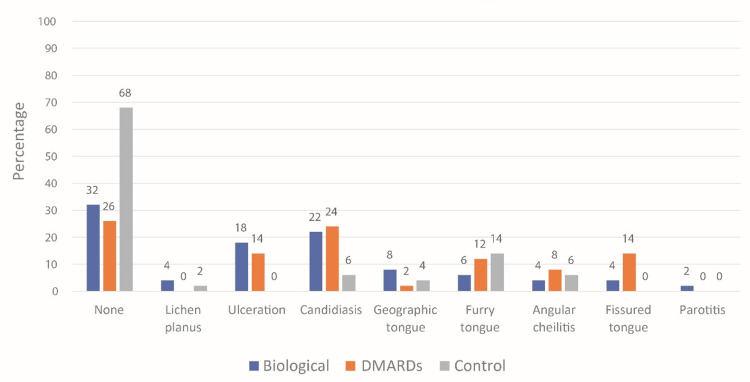
Oral mucosal lesion by treatment type Note: The p-value was not calculated because of insufficient computer memory. After removing the “none” category, the p-value was 0.015 (Fisher’s exact test). DMARD: Disease-modifying anti-rheumatoid drug.

In general, OML are more common among patients of the biological and DMARD groups than the control group. Figure 2 shows that 34 individuals (68%) of the control group had no OML, compared with 16 individuals (32%) of the biological group and 13 individuals (26%) of the DMARD group. The most common lesions in the biological group were candidiasis in 11 individuals (22%), ulceration in nine individuals (18%), and geographic tongue in four individuals (8%). The most common lesions in the DMARD group were candidiasis in 12 individuals (24%), ulceration in seven individuals (14%), fissured tongue in seven individuals (14%), furry tongue in six individuals(12%), and angular cheilitis in four individuals (8%). Other details are shown in Figure 2.

**Figure 3:**
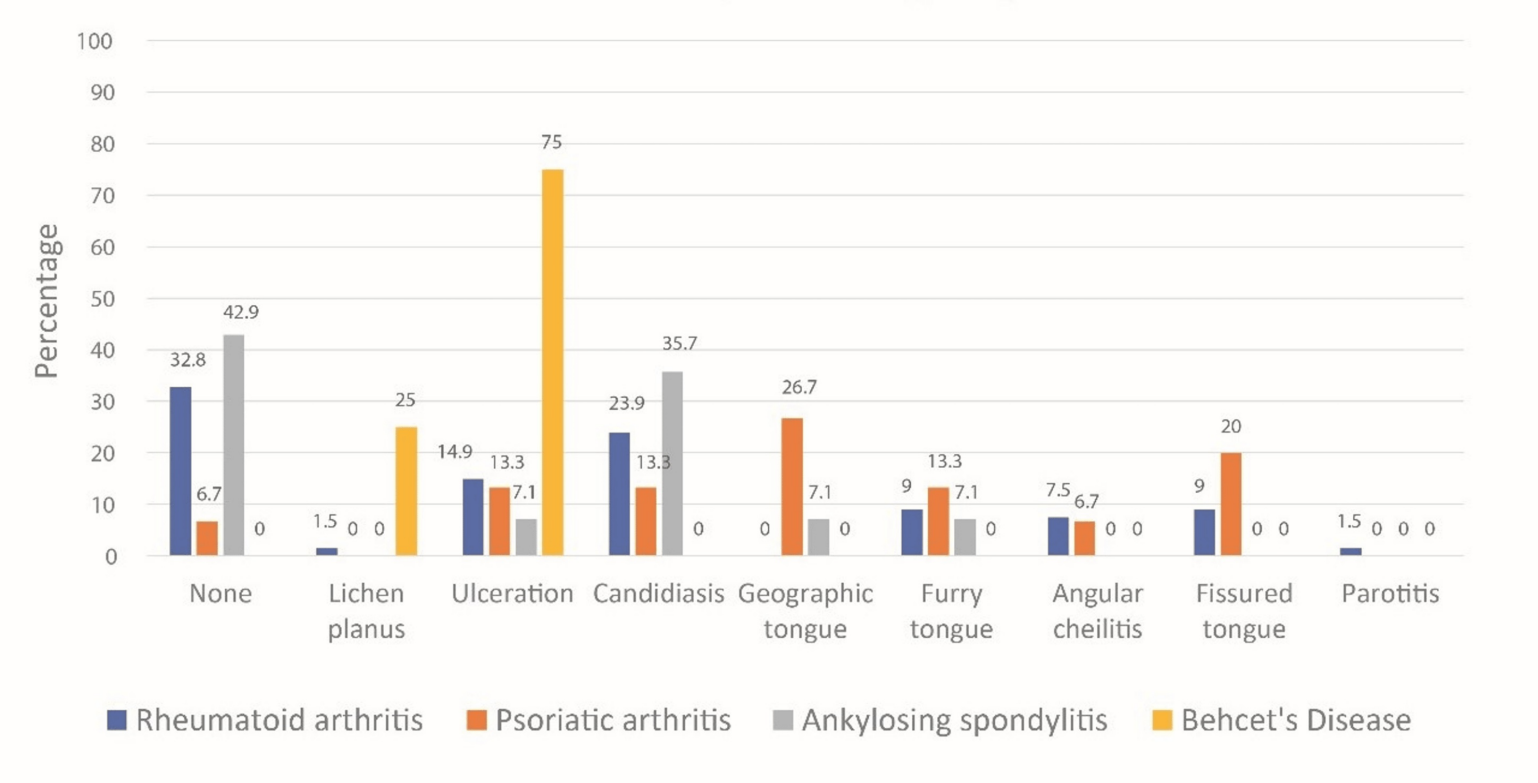
Oral mucosal lesion by rheumatologic diagnosis p = 0.001 (Chi-square test). Note that Fisher’s exact test should be used (but it was not used because of insufficient computer memory).

In each rheumatological condition, the most common OML included lichen planus, leukoplakia, and candidiasis, with the prevalence varying between conditions. For example, lichen planus was more prevalent in patients with systemic lupus erythematosus (SLE), while leukoplakia was more frequently observed in those with RA. These differences highlight the potential impact of disease-specific factors on oral mucosal health, as evident in Figure 3, which shows that some of the OML are more common in one or two rheumatologic conditions than in other conditions. The majority (3, 75%) of patients with Behcet’s disease had ulceration, five (35.7%) of the ankylosing spondylitis patients had candidiasis, four (26.7%) of PA patients had geographic tongue, and three (20%) had fissured tongue. Other details are shown in Figure 3.

## Discussion

This case-control study demonstrates that patients with rheumatologic disease currently on DMARDs or biological therapy at Rizgary Teaching Hospital have evidence of worse OH and OH-related QoL compared to controls. Demographic characteristics with a focus on age, gender, education level, and lifestyle factors among the three distinct groups clarify that education level emerged as a differentiating factor among the groups. A higher percentage of individuals in the control group held a degree compared to the other two groups, suggesting a potential association between education level and overall OH conditions. In contrast, groups on DMARDs and biological therapy had worse overall OH conditions. This finding aligns with the earlier studies confirming that patients with rheumatic diseases show a reduced OH-related QoL [25].

Patients receiving DMARDs and biological therapies had significantly higher levels of dry mouth, worse oral hygiene, more missing teeth, and more decayed teeth than controls. However, the prevalence of dry mouth and the proportion of patients complaining of "very frequent" dry mouth is higher in the group on biological therapy compared to DMARDs.

The elevated instances of xerostomia observed in this study align with prior investigations exploring reduced salivary flow in rheumatologic conditions [12]. Hyposalivation in the oral cavity is linked to various additional oral health issues, including periodontitis, dental caries, oral candidiasis, and poorer OH-related QoL [12].

In this study, LOA, a pivotal indicator of periodontal health, was slightly higher in the cases compared to the control group but was not considered as significant. Earlier investigations on periodontitis in RA and SS demonstrated a higher prevalence of periodontitis when compared to control groups [10,12].

Periodontitis correlates with various risk factors, including unfavorable genetic polymorphisms, smoking, stress, systemic disease, and inadequate oral hygiene [26]. Within the scope of this study, a limited prevalence of smoking was observed in both the control group and the cases. Consequently, the infrequency of smoking contributes to the relatively uncommon occurrence of periodontitis. Because of the poorer oral hygiene in individuals taking DMARDs and biological medications compared to those in the control group, there was a slightly higher occurrence of periodontitis in the former two groups. Inadequate oral hygiene results in the accumulation of dental plaque on teeth, which may undergo calcification over time, resulting in the formation of dental calculus. This process exacerbates the inflammatory response of periodontal tissues, thereby contributing to the development and progression of periodontal disease [26].

The findings of this study underscore the need for routine OH monitoring in rheumatologic patients undergoing DMARD or biologic therapy. Clinicians should consider integrating dental evaluations into the management plans for these patients, with a focus on early detection and treatment of oral infections. Preventive strategies, such as the use of antifungal agents for patients at high risk of oral candidiasis and the promotion of enhanced oral hygiene practices, are recommended. Additionally, future research should aim to explore the long-term effects of these therapies on OH to understand the cumulative risk over time better. In this study, missing teeth among groups on DMARDs and biological therapy were significantly higher compared to the control group. This result confirms the outcome of earlier studies that have demonstrated a correlation between tooth loss and conditions such as RA and SS [27].

The prevalence of OML appears to be higher in groups undergoing biological therapy and DMARDs when compared to the control group. Notably within the biological therapy recipient group, oral candidiasis emerges as the most frequent lesion, succeeded by ulceration and geographic tongue, respectively. In the DMARDs-receiving group, candidiasis similarly prevails as the predominant lesion, followed sequentially by ulceration, fissured tongue, furry tongue, and angular cheilitis. In previous studies, results show that manifestations such as oral ulcers or xerostomia are not specific and very frequent [28]. The reason for the increased cases of candidiasis noted in this study may be attributable to the common adverse effect of increased risk of infection induced by biological therapies and DMARDs including bacterial, viral, and fungal infections [6].

Prior investigations have noted an elevation in the prevalence of OML in psoriasis, with particular emphasis on conditions such as fissured tongue and geographic tongue [29]. Nonetheless, the available evidence has been limited in establishing a definitive correlation between fissured tongue or geographic tongue and the presence of PA and RA. This current study identified four instances of geographic tongue among the groups receiving biological therapy and DMARDs, whereas the control group exhibited only two cases. Additionally, fissured tongue cases were documented in nine instances among those on biological medication and DMARDs, while none were recorded in the control group. This discrepancy may be linked to the potentially inadequately controlled disease activity in patients with RA and PA who were under the influence of biological medication and/or DMARDs in our study. The association of these tongue conditions with RA and PA warrants further investigation to draw conclusive findings.

The disease activity index is a crucial tool for evaluating the degree of disease activity based on clinical presentations and investigations; it is both reliable and valid [20-24]. In this study, the disease activity index was assessed for each patient in the groups receiving biological therapy and DMARDs. The findings show no significant connection between disease activity and OH indicators, such as OML, LOA, and dry mouth. However, a significant correlation was observed in DMFT among patients with severe and moderate disease activity compared to those in remission. This observation may be because many patients on biological therapy and DMARDs have their disease under control. Other studies assessing the correlation between oral manifestations in RA patients in Baghdad undergoing different treatments and disease activity similarity have shown no significant connection between disease activity and oral health [30].

The strengths of this study encompass a detailed objective OH examination conducted by proficient dentists, along with the utilization of a comprehensive and standardized OH questionnaire. The groups undergoing biological therapy and DMARDs comprised individuals with rheumatological diseases such as RA, PA, ankylosing spondylitis (AS), and Behcet’s disease (BD). The sample size was sufficiently large to highlight variations compared to the control groups. Furthermore, another strength of this study is the comprehensive subjective questionnaire assessing dry mouth [18]. Current methods primarily rely on subjective questioning and complex investigations like sialometry. The perception of dry mouth does not necessarily correlate with reduced saliva production, and objective measurement by sialometry has been rare in studies investigating OH in connective tissue diseases.

Additionally, it is important to consider the differences in the prevalence of OML across various rheumatological conditions. These differences may be attributed to variations in disease pathophysiology, immune system involvement, and the specific medications used for treatment. For example, the immunosuppressive effects of DMARDs may contribute to a higher incidence of oral candidiasis, whereas biologics targeting specific inflammatory pathways may result in varying impacts on oral tissues. Further research is needed to explore these mechanisms in greater detail. When comparing our findings to prior studies, similarities were observed in the increased prevalence of OML in patients with rheumatological diseases, particularly those undergoing immunosuppressive therapy. However, discrepancies in the incidence of periodontal disease between studies may be attributed to differences in sample populations, medication regimens, and oral hygiene practices. Our study highlights the role of medication, particularly DMARDs and biologics, in altering OH outcomes through immunosuppressive and anti-inflammatory mechanisms. These drugs may increase susceptibility to oral infections while reducing inflammation that exacerbates periodontal disease.

Other constraints include the absence of additional periodontal assessments, such as periodontal probing depth and bleeding on probing. These assessments were deemed impractical due to their potential discomfort and invasiveness, especially in a busy outpatient clinic lacking access to a dental chair or dental assistant. However, evaluating LOA using the CPI system is designed to provide an estimate of the lifetime accumulated destruction of periodontal attachment, allowing comparisons between different groups. Our findings underscore the importance of integrating oral healthcare into the routine management of patients with rheumatological diseases. Regular OH assessments, particularly for patients receiving long-term biologic or DMARD therapy, should be considered a critical component of care. Collaborative efforts between rheumatologists and dental professionals may improve the detection and management of OH complications in these patients.

The limitation of our study is the broad spectrum of rheumatological conditions included, which may introduce variability in OH outcomes. One potential bias in our study is the variation in baseline health and lifestyle factors, such as dental hygiene practices, medication adherence, and access to dental care. These factors could have influenced the OH outcomes observed and should be controlled for in future research. Additionally, the relatively short follow-up period of six months post-surgery limits our ability to assess long-term OH outcomes. Longer-term studies are needed to evaluate the durability of the observed improvements in OH.

Future research should focus on the long-term OH outcomes of patients with rheumatological diseases, particularly those on newer biological therapies. Longitudinal studies with extended follow-up periods would provide valuable insights into the lasting effects of these treatments on OH. Additionally, personalized dental interventions tailored to the specific needs of patients with different rheumatological conditions could be explored to improve OH outcomes.

## Conclusions

In conclusion, the findings indicate that OH issues, such as OML and dry mouth, are more prevalent among patients receiving DMARDs or biological therapy treatments compared to a control group. Notably, the DMARD group exhibited higher decayed and missing teeth indices, while the control group had the highest filled teeth scores. Furthermore, disease severity was associated with higher DMFT scores, underscoring the significant impact of rheumatologic disease activity on OH. Additionally, OML patients with rheumatologic diseases are more susceptible to candidiasis. These results highlight the necessity for integrated oral healthcare strategies in the management of patients with rheumatologic diseases. This study highlights the significant OH challenges faced by patients with rheumatological diseases, particularly in the context of long-term DMARD and biological therapy. Findings emphasize the need for regular OH assessments and the integration of dental care into the management plans of these patients. Collaborative care between rheumatologists and dentists can help mitigate OH complications and improve overall patient outcomes. Future research should focus on longitudinal studies to further elucidate the relationship between rheumatological disease, treatment regimens, and OH outcomes. Additionally, studies examining newer biological therapies and personalized dental interventions tailored to specific rheumatological conditions will be critical in advancing the management of these patients.
